# The direct superior approach versus posterior approach for total hip arthroplasty: study protocol for a prospective double-blinded randomised control trial

**DOI:** 10.1186/s13063-020-04484-y

**Published:** 2020-06-19

**Authors:** Babar Kayani, Sujith Konan, Jenni Tahmassebi, Atif Ayuob, Fares S. Haddad

**Affiliations:** Department of Trauma and Orthopaedic Surgery, University College Hospital, 235 Euston Road, Fitzrovia, London, NW1 2BU UK

## Abstract

**Background:**

The direct superior approach (DSA) is a minimally invasive modification of the posterior approach (PA) that preserves the iliotibial band and short external rotators except for the piriformis or conjoint tendon during total hip arthroplasty (THA). The objective of this study is to compare patient satisfaction, functional outcomes, accuracy of implant positioning, component stability, gait, cost-effectiveness, and complications in the DSA versus PA for THA.

**Methods and analysis:**

This prospective double-blinded randomised control trial will include 80 patients with symptomatic hip osteoarthritis undergoing primary THA. Following informed consent, patients will be randomised to THA using the PA (control group) or DSA (investigation group) at a ratio of 1:1 using an online random number generator. Blinded observers will review patients at regular intervals for 2 years after surgery to record predefined study outcomes relating to postoperative rehabilitation, clinical progress, functional outcomes, accuracy of implant positioning, gait analysis on force plate treadmill, implant migration with radiosteriometric analysis, cost-effectiveness, and complications. A superiority study design will be used to evaluate whether the DSA provides improved outcomes compared to the PA for THA. Evaluation of study outcomes in DSA and PA will be used to quantify and draw inferences on differences in the efficacy of treatment between the two groups. Intention-to-treat and per-protocol population analysis will be undertaken. The following statistical methods will be employed to analyse the data: descriptive statistics, independent *t* test, paired *t* test, analysis of variance, Fisher exact test, chi-square test, and graphical displays. Ethical approval was obtained from the London-Fulham Research Ethics Committee, UK. The study is sponsored by University College London, UK.

**Discussion:**

This study compares a comprehensive and robust range of clinical, functional, and radiological outcomes in THA performed using the PA versus DSA. The findings of this study will provide an improved understanding of the differences in the PA versus DSA for THA with respect to patient satisfaction, functional outcomes, implant survivorship, gait, cost-effectiveness, and complications.

**Trial registration:**

ClinicalTrials.gov, NCT04191993. Registered on 10 December 2019

## Background

Total hip arthroplasty (THA) is an effective procedure for relieving pain, restoring function, and improving the quality of life in patients with end-stage hip osteoarthritis [2–5, 7, 16, 20]. Analysis of joint registry data from the UK, Sweden, and New Zealand has shown that the posterior approach (PA) is the most commonly used approach for THA [16]. The main advantages of the PA for THA are that it preserves the abductor mechanism, reduces intraoperative blood loss, and decreases the risk of heterotrophic ossification compared to the anterolateral and direct lateral approaches [10, 14, 16]. However, the PA is associated with increased risk of sciatic nerve injury during dissection, bleeding from the inferior gluteal artery as it leaves the pelvis below the piriformis, and increased risk of dislocation compared to these other approaches [6, 8, 11, 25]. Evolution in minimally invasive surgery and recent innovations in surgical instrumentation have led to the development of the direct superior approach (DSA), which is a modification of the PA that preserves the iliotibial band and the short external rotators except for the piriformis or conjoint tendon [17, 19, 21]. Conceptually, improved preservation of this periarticular soft tissue envelope may help to reduce postoperative pain, improve functional recovery, and better restore native hip biomechanics [13, 18].

Existing studies on the DSA for THA are case-controlled trials or case series with limited data on validated functional outcomes or clinically significant radiological outcomes [17, 19, 21]. Nam et al. conducted a prospective non-randomised trial comparing THA performed using 196 PAs versus 42 DSAs at three separate treatment centres [17]. The authors reported there was no difference in moderate to severe pain over the greater trochanter, anterior thigh, and lateral thigh between the two approaches at a minimum 1-year follow-up. Patients undergoing the PA had improved University of California at Los Angeles hip (UCLA) scores compared to those undergoing the DSA at 1-year follow-up. However, this study included different operating surgeons within three healthcare institutions, and the PA was performed through a modified mini-incision in patients that were younger and more active than the DSA group. Roger and Hill retrospectively reviewed outcomes in a case series of 135 patients undergoing the DSA for THA and reported good postoperative functional outcomes as assessed using the Harris hip score at 1-year follow-up [21]. Mean acetabular cup abduction was 41° (range 21–49°), and mean acetabular cup anteversion was 21° (range 15–27°). Femoral implant positioning greater than 2° varus and valgus was observed in 4% and 2% of patients respectively. This study was a retrospective study with no control group and heterogeneity in postoperative follow-up times.

Penenberg et al. followed 250 patients undergoing the DSA for THA and found mean Harris hip scores improved from 47.71 preoperatively to 95.6 within 3 to 6 months postoperatively [19]. Femoral implant positioning was within 2° of the planned position in 97% of patients with mean acetabular cup abduction of 42° (range 30 to 55°) and acetabular cup anteversion of 31° (range 22 to 40°) in all study patients. This was a retrospective study with limited follow-up, no control group, patients and clinicians were not blinded, and statistical analysis for assessing the significance of study outcomes was not performed. Amanatullah et al. assessed periarticular muscle injury in eight cadaveric specimens in which the direct anterior approach was performed on one side and the DSA for THA on the contralateral side [1]. The DSA was associated with reduced iatrogenic injury to the gluteus minimus muscle, gluteus minimus tendon, tensor fascia lata, and rectus femoris compared to the direct anterior approach. This study had a small sample size and iatrogenic soft tissue injury was graded in cadaveric specimens, and therefore, it remains unknown how the observed differences in muscle injury translate to clinical outcomes between the two treatment groups.

There remains a paucity of high-quality evidence comparing clinical, functional, and radiological outcomes in the PA versus DSA for THA. Further, it remains unknown how differences in muscle preservation in the DSA and PA translate to postoperative pain scores between the two treatment groups. It is possible to improve on existing studies by assessing a more comprehensive and robust range of outcome measures, prospectively randomising patients to their respective treatment groups, standardising the surgical techniques within each group, using the same implant designs and postoperative rehabilitation protocol in all study patients, and blinding patients and observers recording study outcomes. Radiosteriometric analysis (RSA) will also be used to assess implant micromotion, which correlates with component loosening and provides prognostic information on long-term implant survivorship [22–24]. The findings of this study will provide an improved understanding of the differences in the PA versus DSA for THA with respect to patient satisfaction, functional outcomes, accuracy of implant positioning, component survivorship, cost-effectiveness, and complications.

## Methods/design

### Objectives

The primary objective of this study is to compare postoperative pain scores on the visual analogue scale (VAS) in the PA versus DSA for THA. The study hypothesis is that the DSA will reduce periarticular soft tissue dissection that will translate to reduced postoperative pain scores on the VAS compared to the PA for THA.

The secondary objectives are to compare the following outcomes between the two treatment groups:

1. Surgical efficiency
2. Postoperative functional rehabilitation
3. Patient satisfaction
4. Functional outcomes
5. Quality of life
6. Muscle group strength
7. Range of motion
8. Accuracy of restoring planned hip biomechanics
9. Accuracy of achieving planned component positioning
10. Implant stability
11. Gait analysis
12. Resource use and cost-effectiveness
13. Complications

### Trial design

**Fig. 1:**
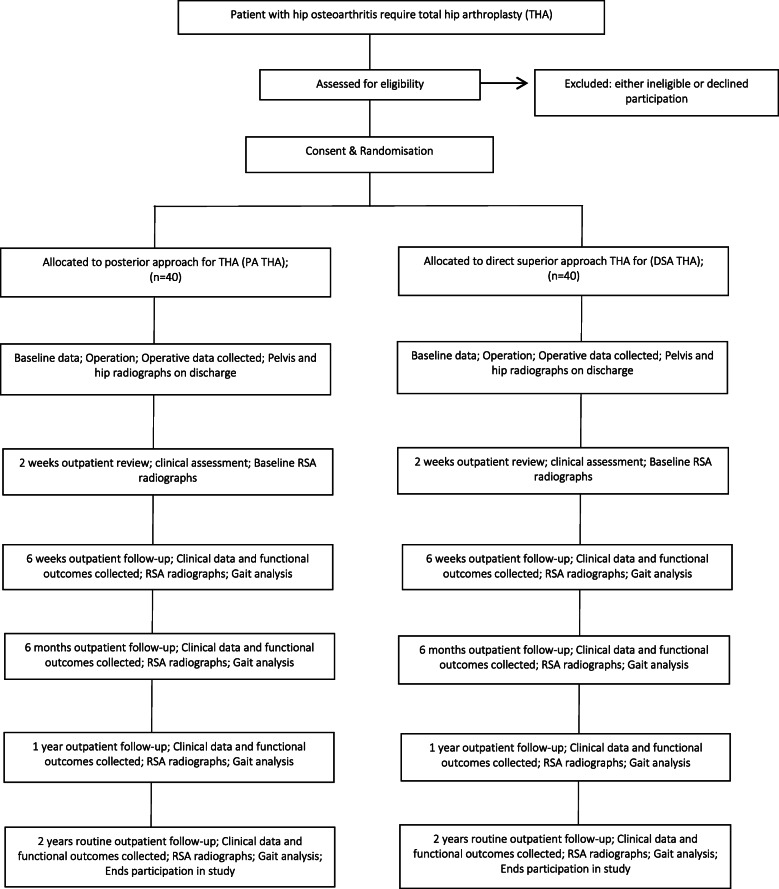
Patient enrolment flow chart

**Fig. 2:**
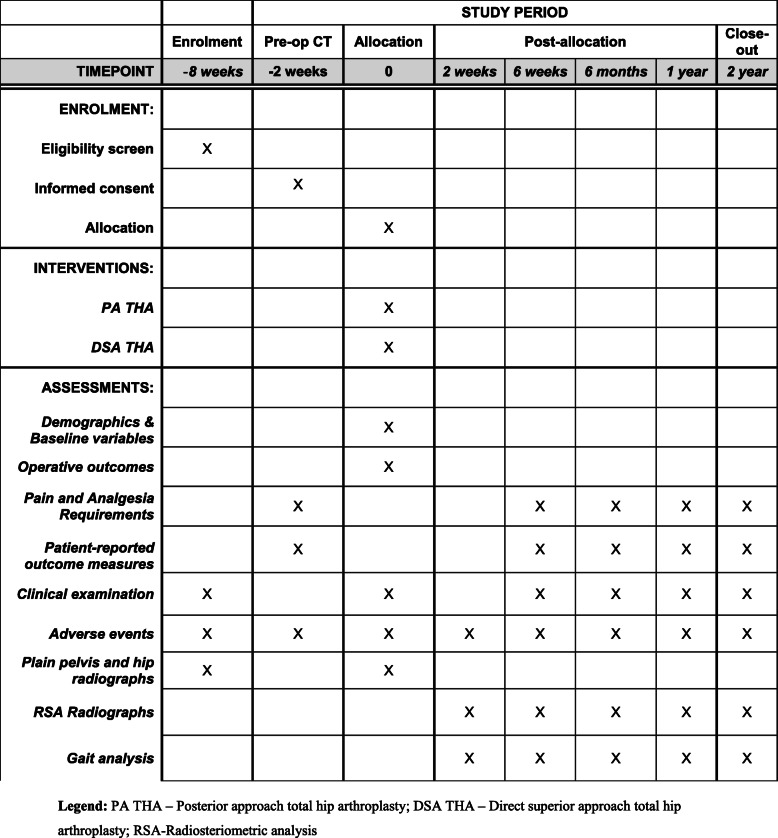
Schedule of enrolment, interventions, and assessments for all study patients. PA THA posterior approach total hip arthroplasty, DSA THA direct superior approach total hip arthroplasty, RSA radiosteriometric analysis

This study is a prospective, single-centre, double-blinded, randomised control trial. The study will be undertaken in the Department of Trauma and Orthopaedics, University College Hospital, 235 Euston Road, Bloomsbury, London NW1 2BU, UK. The study will include 80 patients randomly allocated to either PA (control group) or DSA (investigation group) for THA. The study commenced patient recruitment in June 2018 and is expected to complete patient recruitment in December 2020. All patients will be followed up for 2 years after surgery, and therefore, the anticipated completion date for the study is December 2022. The study is sponsored by University College London, UK. The patient enrolment flowchart is presented in Fig. 1. The schedule of enrolment, interventions, and assessments for all study patients is shown in Fig. 2.

### Eligibility criteria

The inclusion criteria for this study are as follows: the patient has symptomatic hip osteoarthritis requiring primary THA; the patient is fit for surgical intervention following review by a surgeon and anaesthetist; the is patient aged between 18 and 80 years at the time of surgery; the patient is able to give informed consent and agrees to comply with the postoperative review programme; and the patient has sufficient mobility to attend follow-up clinics. The exclusion criteria for this study are as follows: patient is undergoing revision surgery or second-stage THA; patients in whom the planned hip biomechanics are in a different position to the contralateral hip (e.g. developmental dysplasia of the hip or protrusio acetabuli); patient is not suitable to have the planned study implants (e.g. requiring dual mobility component or cemented implants); patient is immobile or has another neurological condition affecting musculoskeletal function; patient is already enrolled on another concurrent clinical trial; patient is unable or unwilling to sign the informed consent form specific to this study; and patient is unable to attend the study follow-up programme. Importantly, patients that do not receive the planned study implants (e.g. intraoperative assessment showing poor bone quality or bone loss necessitating cemented implants for THA) will be excluded from the study.

### Recruitment

Patients will be recruited from the orthopaedic outpatient clinic at University College Hospital, London, UK. All patients will be screened by the clinical team (orthopaedic consultant surgeon, clinical research fellow, and orthopaedic registrar) for study participation based on the predefined inclusion and exclusion criteria listed above. Patients that fulfil the eligibility criteria and express an interest to participate in the study will be provided with an ethics committee-approved patient information sheet. This provides details about the study treatment, follow-up, and contact details for further information. All members of the clinical team are familiar with the study and will address any preliminary questions about the study. Details of those patients expressing an interest to participate in the study will be recorded in the patient contact form and forwarded to the research physiotherapist. The research physiotherapist will phone the patient 4 weeks after this consultation to discuss any further questions and confirm if the patient would like to participate in the study.

### Consent

Informed consent will be obtained by the chief investigator or principal investigator when the patient attends for preoperative assessment. This is 6 weeks after the outpatient consultation for agreement to THA and 2 weeks before surgery. It is important to the data collection scheme that patients are able to follow commands, read, and interpret questions via questionnaires. For those who cannot hear, read, or understand English, an interpreter will be provided. Identical preoperative imaging modalities for surgical planning will be used in both treatment groups.

### Allocation

After informed consent has been obtained, the research physiotherapist will randomise the patient into one of the two treatment groups using an online random number generator (www.random.org). A number from 1 to 80 can be randomly generated and will allocate a patient to one of the two arms of the study: 1–40 inclusive for the control group and 41–80 inclusive for the investigation group. The research physiotherapist will perform the randomisation procedure and store the designated treatment group for each patient on a password-encrypted file on the hospital computer. The operating surgeon will have this information communicated to them on the morning of surgery.

### Preoperative imaging

All patients will undergo preoperative imaging with plain pelvic and hip radiographs. In both treatment groups, pelvic radiographs will be exported onto Traumacad software (Traumacad, Petach-Tikva, Israel) to template optimal implant positioning and sizes for achieving the planned bone coverage, component version and inclination, horizontal and vertical centres of rotation, acetabular and femoral offset, and leg-length correction. All preoperative templating will be undertaken by the operating surgeon 2 weeks prior to surgery. Preoperative pain scores on the VAS, analgesia requirements, and functional outcome scores (Fig. 2) will also be collected preoperatively as baseline values.

### Surgical intervention

**Fig. 3:**
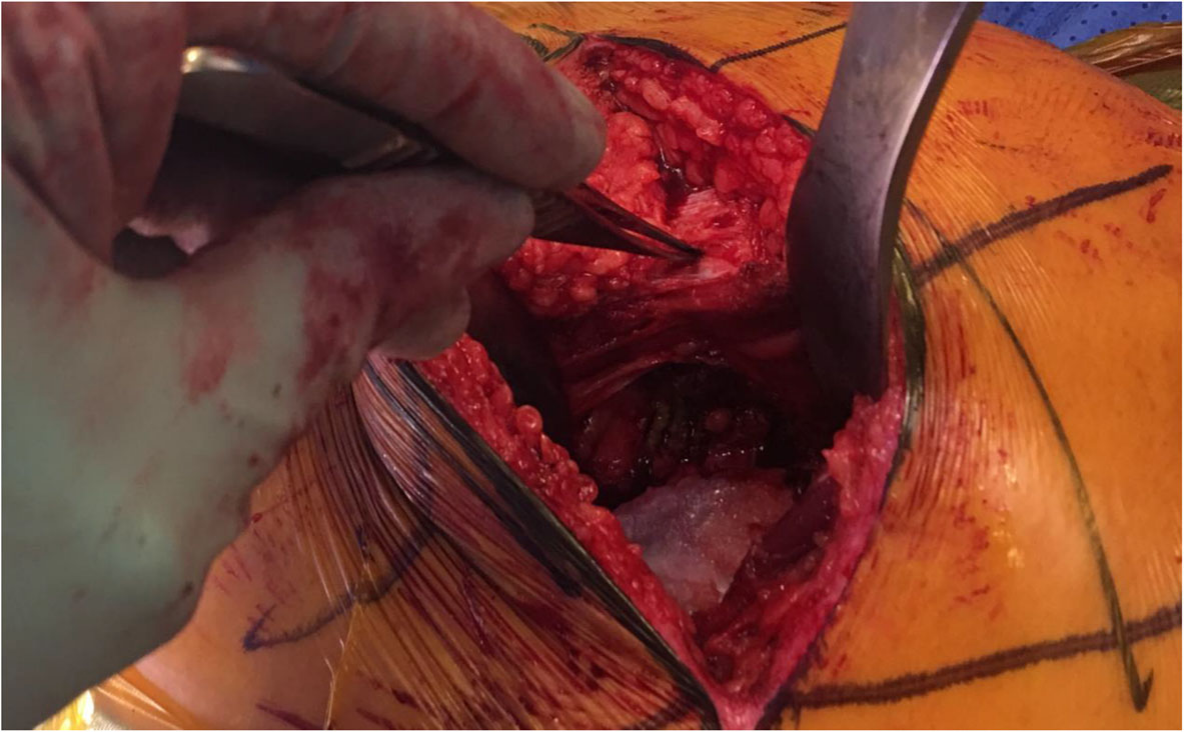
intraoperative photo showing the preservation of the iliotibial band.

All operative procedures will be performed by one of two consultant orthopaedic surgeons that are fully trained and experienced with both the PA and DSA for THA. In both treatment groups, the patient will be placed in the lateral decubitus position and a curved incision performed behind the posterior edge of the greater trochanter extending distally towards the shaft of the femur. This initial incision will be 8–10 cm in length but can later be extended as required. The fascia lata will be incised along the length of the incision and the fibres of the gluteus maximus split along the line of the incision to expose the underlying pericapsular fat. Retractors will be used to retract the gluteus medius anteriorly and the soft tissue along the posterior border of the proximal femur inferiorly. The pericapsular fat will be removed with diathermy and the plane between the gluteus medius and gluteus minimus muscle developed. The hip joint will then be internally rotated to place the short external rotators on stretch. In the PA, diathermy will be used to detach the external rotators (including piriformis) close to their femoral insertion. In the DSA, caution will be taken to identify and protect the iliotibial band during dissection (Fig. 3) [12, 19, 21]. A retractor will be placed directly under the obturator internus to protect the inferior gemellus muscle below. The piriformis (or conjoint tendon) will be detached as close to their femoral insertions as possible but the remaining external rotators preserved. The capsulotomy will start at the distal, inferolateral aspect of the wound and extend proximally and posteromedially towards the superior acetabular margin. The capsule will be elevated subperiosteally to create superior and inferior capsular flaps. The retractors will then be repositioned inside the capsule and the hip dislocated posteriorly by flexion, adduction, and internal rotation.

The femoral osteotomy site will be marked using the templated measurements from the greater and lesser trochanters with the femoral neck cutting guide in place. An oscillating saw will be used to perform the osteotomy with Hohmann retractors protecting the surrounding soft tissues. The femoral osteotomy will be performed with the saw blade 45° to the femoral shaft and in the plane of the tibia. Sharp Hohman retractors will be positioned over the anterior wall to lever the femur anteriorly and under the transverse acetabular ligament to expose the whole acetabulum for preparation. Soft tissues overhanging the acetabular circumference will be excised. Osteophytes will also be excised with an osteotome and the medial wall visualised. Hemispherical reamers will be used to remove the residual acetabular cartilage and expose the underlying subchondral bone. In the PA, this will be undertaken using sequentially larger straight reamers until the medial wall is reached. In the DSA, medial acetabular wall reaming will initially be undertaken using a straight acetabular reamer and then sequentially larger angled reamers until the medial wall is reached [19, 21]. In both groups, an external alignment guide will be attached to the cutting-edge reamer handle and acetabular impactor to improve the accuracy of acetabular cup positioning. Acetabular cup positioning will be guided by the preoperative surgical plan and transverse acetabular ligament to match the patient’s native acetabular cup inclination and version within Lewinnek’s safe zones (inclination 30–50°, anteversion 5–25°) [15]. Any residual osteophytes will be removed at this stage using an osteotome and bone nibbler, and the trial prosthesis inserted to ensure satisfactory coverage and stability. Line-to-line technique for acetabular implantation will be used with implantation of the acetabular cup that is the same size as the last reamer used, and this will be augmented with two acetabular screws. The leg will then be placed into 40° flexion, 40° internal rotation, and 40° abduction to visualise the femoral osteotomy site. A femoral elevator will be inserted under the anterior femoral neck to elevate and expose the proximal femur, and a box chisel used to remove bone from the posterolateral femoral neck. The femur will be prepared using a rasp and sequential broaching until maximum cortical contact in the mediolateral dimension is achieved. The femur will be prepared and implanted with 10–20° anteversion. Trial femoral heads will be applied to check hip joint stability, offset, soft tissue tension, and leg-length discrepancy prior to definitive selection of the femoral head. The final femoral stem will then be implanted, femoral head applied to the taper, and hip joint reduced. The capsule and external rotators will be repaired back to the femur and a layered closure of the fascia, subcutaneous tissue, and skin performed.

All study patients will receive the Accolade II femoral stem (Stryker Ltd, Mahwah, NJ, USA) and the trident acetabular shell (Stryker Ltd, Mahwah, NJ, USA). Patients in both treatment groups will undergo the same postoperative rehabilitation programme prior to discharge. All patients will receive postoperative patient-controlled analgesia (PCA) with the background intravenous morphine infusion rate set at 0.5 mg/h, bolus dose of 2 mg, and lockout period of 10 min. Additional oral paracetamol and ibuprofen may be administered by the nursing staff if requested by the patient. The PCA will be stopped 24 h after surgery and converted to an oral regimen of regular paracetamol, ibuprofen, and dihydrocodeine, with oral morphine available for breakthrough pain.

### Outcomes

All study patients will undergo review by two blinded observers (one orthopaedic registrar and one clinical research fellow) at 2 weeks, 6 weeks, 6 months, 1 year, and 2 years following surgery. During these follow-up times, predefined clinical, functional, and radiological outcomes will be recorded by these observers using case report forms (CRFs). The following outcomes will be recorded in all study patients:

1. Operating time (minutes)
2. Time to hospital discharge (hours)
3. Pain scores on the VAS and analgesia requirements preoperatively, during the inpatient admission, and postoperatively at 6 weeks, 6 months, 1 year, and 2 years
4. Patient satisfaction as assessed using the Musculoskeletal Outcomes Data Evaluation and Management System (MODEMS) preoperatively and postoperatively at 6 weeks, 6 months, 1 year, and 2 years following surgery [9]
5. Patient-reported outcome measures including Oxford hip score (OHS), Harris hip score (HSS), hip disability and osteoarthritis outcome score (HOOS), University College Hospital hip (UCH) score, and Western Ontario and McMaster Universities Arthritis Index (WOMAC) preoperatively and postoperatively at 6 weeks, 6 months, 1 year, and 2 years following surgery
6. Health-related quality of life as measured using European Quality of Life questionnaire with 5 dimensions for adults (EQ-5D) preoperatively and postoperatively at 6 weeks, 6 months, 1 year, and 2 years
7. Accuracy of achieving the planned implant positioning and hip biomechanics as assessed using plain pelvic and hip radiographs preoperatively and postoperatively prior to discharge
8. Mobilisation distance (metres) and use of mobility aids preoperatively and postoperatively at 6 weeks, 6 months, 1 year, and 2 years
9. Range of movement (degrees) in hip joint preoperatively and postoperatively at 6 weeks, 6 months, 1 year, and 2 years
10. Muscle group strength testing in hip joint preoperatively and postoperatively at 6 weeks, 6 months, 1 year, and 2 years as measured using a dynamometer
11. Gait analysis performed postoperatively at 6 weeks, 6 months, 1 year, and 2 years as assessed using force plate treadmill testing
12. Femoral and acetabular implant early migration as assessed using RSA performed postoperatively at 2 weeks, 6 weeks, 6 months, 1 year, and 2 years
13. Resource use and cost-effectiveness including comparisons between the two treatment groups relating to operating time, theatre efficiency, equipment and sterilisation costs, analgesia requirements, inpatient rehabilitation, time to discharge, outpatient follow-up, functional outcomes, quality of life, return to work, additional imaging costs, and complications with their respective treatments
14. Complications

### Blinding

All patients and clinical staff recording postoperative study outcomes will remain blinded to the treatment group. Study patients will be identifiable with a unique study number. Only the research physiotherapist will have the key to identify individual patients and their respective treatment arm. Any documents related to the study will be archived directly at the study site by the research physiotherapist within a locked filing cabinet in a locked research office. This office has swipe card access with onsite security and 24-h CCTV surveillance. Patient data will be logged electronically using each patient’s unique identification number with computer software on an encrypted, password-protected research computer.

### Sample size

Prior to commencement of the study, a sample size of 80 patients (40 patients in each treatment arm) was selected to achieve a power of 80% (1 – *β*) for assessing differences in pain scores on the visual analogue scale at 24 h after surgery between the two treatment groups, using an effect size of 0.67, alpha value of 0.05, and accounting for 10% sample size attrition rate during the 2-year follow-up period [17, 20]. A sample size attrition rate of 10% was selected based on previous randomised controlled studies performed in our treatment centre.

### Statistical analysis

The analysis of the per-protocol population will be considered the primary analysis. The differences between the PA and DSA groups will be analysed by calculating the difference from baseline, per patient, and a two-sided confidence interval for the difference between the changes from baseline values will be calculated. This confidence interval will cover the true difference in the percentage change from baseline with a probability of 95%. The following statistical methods will be employed to analyse the data: descriptive statistics, independent *t* test, paired *t* test, analysis of variance, Fisher exact test, chi-square test, and graphical displays. Assumptions of normality will be tested with the D’Agostino test. Assumptions of homogeneity of variance will be tested with Levene’s test. If the distributional assumptions are (severely) violated, non-parametric techniques, such as Mann-Whitney’s test will be employed. In the event that the DSA is converted to PA intraoperatively, analysis will be performed using the intention-to-treat population and the treatment actually received by the patients. Intraoperative conversion from DSA THA to PA THA will be documented and presented and published as part of the study. Statistical significance is set at a *p* value < 0.05 for all analyses and all statistical analysis will be performed using SPSS software version 26 (SPSS Inc., Chicago, IL, USA).

### Adverse events

Adverse events are defined as any untoward medical occurrence in a patient or study participant, which does not necessarily have a causal relationship with the procedure involved. A serious adverse event (SAE) is an adverse event that results in hospitalisation or prolongation of existing hospitalisation, persistent or significant disability or incapacity, life-threatening clinical sequelae, or death. All SAEs during the protocol treatment will be reported directly to the sponsor using the SAE web form. The chief investigator will also assess the SAE for severity, causality, seriousness, and expectedness using pre-existing criteria provided by the sponsor and inform the Data Safety Monitoring Board (DSMB) within 3 days of the initial observation of the event. The protocol treatment period is defined as the period from the day that the first study patient is recruited into the trial to the day that the final study patient has completed the 2-year follow-up. The chief investigator will also inform the London-Fulham Research Ethics Committee and local Health Research Authority within 3 days of the SAE taking place. Safety aspects of the study are closely monitored by the sponsor and DSMB using unblinded data for its judgment. In cases where the SAE arises due to a problem with the study implants, Stryker Limited will also be notified within 2 days of the event taking place. The chief investigator will record the following: onset date, complete description of the event, severity, duration, action taken, and outcome for each SAE. The chief investigator will also provide regular updates of all SAEs to the London-Fulham Research Ethics Committee, local Health Research Authority, DSMB, and sponsor.

### Data management

Onsite monitoring visits shall occur throughout the course of the clinical study by the chief investigator. The chief investigator shall permit and assist the sponsor (should they chose to monitor the study) to carry out verification of all study forms against data in the source documents, which shall occur as per the departmental policy for undertaking such activities. University College Hospital recognises that there is an obligation to archive study-related documents at the end of the study. The study master file will be archived at University College London in accordance with the University College Hospital Standard Operating Procedure for Archiving of Investigator Site File (ISF) and Pharmacy Site File (PSF). It will be archived for a minimum of 5 years from the study end, and no longer than 30 years from the study end.

### End of protocol treatment

Reasons for going off study protocol include:

- Completion of the last follow-up visit 2 years after surgery
- Patient non-compliance or withdrawal (the reason for discontinuation will be recorded in the case report form)
- Intercurrent death

All patients included in this study are free to withdraw from the study at any time without compromise to their future treatment. On withdrawal, patients will revert to the standard follow-up regimen for routine (non-study) THA at the study site. The end of the study form will be completed and the reason for withdrawal documented. This form will also be completed if the patient is lost to follow-up or dies during the course of the study. Data to the point of discontinuation will be used for analysis.

### Monitoring

The chief investigator will monitor the progress of the clinical study in the form of monthly research meetings for those involved in the trial. The chief investigator will be responsible for day-to-day monitoring and management of the study. The UCLH/UCL/Joint Research Office, on behalf of UCL as the sponsor, will monitor and conduct random audits on a selection of studies in its clinical research portfolio. Monitoring and auditing will be conducted in accordance with the Department of Health Research Governance Framework for Health & Social Care (April 2005) and in accordance with the sponsor’s monitoring and audit policies and procedures. As per the protocol, the principal investigator will email the sponsor twice yearly with the following: delegation log, adverse event log, deviation log, and any annual progress reports sent to the Ethics committee.

### Peer review

The study protocol has undergone independent external peer reviewer. The suggestions and recommendations for improvement to the study design were implemented. The reviewers, sponsor, and London-Fulham Research Ethics Committee reviewed the revised protocol documents and confirmed that all queries and suggestions had been fully addressed.

## Discussion

THA is an effective procedure for relieving pain, restoring function, and improving the quality of life in patients with end-stage hip osteoarthritis. The surgical approach in THA is important as it influences postoperative gait, hip stability, and muscle function [2–5, 7, 16, 20]. The DSA is a minimally invasive modification of the PA that preserves the iliotibial band and short external rotators except for the piriformis or conjoint tendon during THA [17, 19, 21]. This prospective double-blinded randomised control trial will include 80 patients with symptomatic hip osteoarthritis undergoing primary THA. Following informed consent, patients will be randomised to undergo THA using the PA (control group) or DSA (investigation group) at a ratio of 1:1 using an online random number generator. Blinded observers will review patients at regular intervals for 2 years after surgery to record predefined study outcomes relating to postoperative rehabilitation, clinical progress, functional outcomes, accuracy of achieving planned implant positioning and hip biomechanics, cost-effectiveness, and complications. Gait analysis will be undertaken using force plate treadmills and implant stability assessed using RSA [22–24]. The findings of this study will provide an improved understanding of differences in the PA versus DSA for THA with respect to patient satisfaction, functional outcomes, implant position, implant survivorship, gait, cost-effectiveness, and complications.

## Trial status

Protocol: version 1.0; date 01 September 2017

Patient recruitment date: 1 June 2018

Estimated completion of recruitment date: 1 December 2020

Estimated completion of final follow-up: 1 December 2022

## Data Availability

The datasets used and/or analysed during the current study are available from the corresponding author on reasonable request.

## Abbreviations

CRF: Case report form
DSA: Direct superior approach
DSMB: Data Safety Monitoring Board
EQ-5D: European Quality of Life questionnaire with 5 dimensions for adults
HHS: Harris hip score
HOOS: Hip disability and osteoarthritis outcome score
OHS: Oxford hip score
PA THA: Posterior approach
SAE: Serious adverse event
SF-12: Short form health survey of 12 items
WOMAC: Western Ontario and McMaster Universities Arthritis Index
